# Solitary Crossed Renal Ectopia: Concurrence of Posterior Urethral Valve and Hypospadias

**DOI:** 10.1155/2015/748139

**Published:** 2015-06-18

**Authors:** Amin Bagheri, Reza Khorramirouz, Sorena Keihani, Mehdi Fareghi, Abdol-Mohammad Kajbafzadeh

**Affiliations:** Pediatric Urology Research Center, Pediatrics Center of Excellence, Children's Hospital Medical Center, Tehran University of Medical Sciences, Tehran 1419433151, Iran

## Abstract

Solitary crossed renal ectopia (SCRE) represents an exceedingly rare congenital disorder. Although skeletal and genitourinary abnormalities most commonly accompany this condition, vesicoureteral reflux (VUR) has been described in only a few cases. Here, we present two unique cases of SCRE complicated by high-grade VUR concomitant with posterior urethral valve in one case and hypospadias in the other one. We also provide a brief review of the literature on this subject.

## 1. Introduction

Solitary crossed renal ectopia (SCRE) is a rare congenital anomaly with an estimated prevalence of 1 in 1,500,000 [1]. A combination of unilateral renal agenesis and contralateral renal ectopia that crosses the midline leads to SCRE. Mostly asymptomatic, SCRE often remains undiagnosed or presents a diagnostic challenge as an incidental finding during routine perinatal ultrasound, during screening imaging studies, or at autopsy [2].

Although several genitourinary and skeletal abnormalities may accompany SCRE, concomitant vesicoureteral reflux (VUR) is rarely reported [3, 4]. Moreover, the concurrence of posterior urethral valve (PUV) with these conditions has not been previously reported. Hypospadias is also rare in context of SCRE [4, 5]. Hereby, we report two unique cases of SCRE presenting with VUR complicated by hypospadias (Case 1) and PUV (Case 2) and also provide a brief literature review.

## 2. Case 1

**Figure 1:**
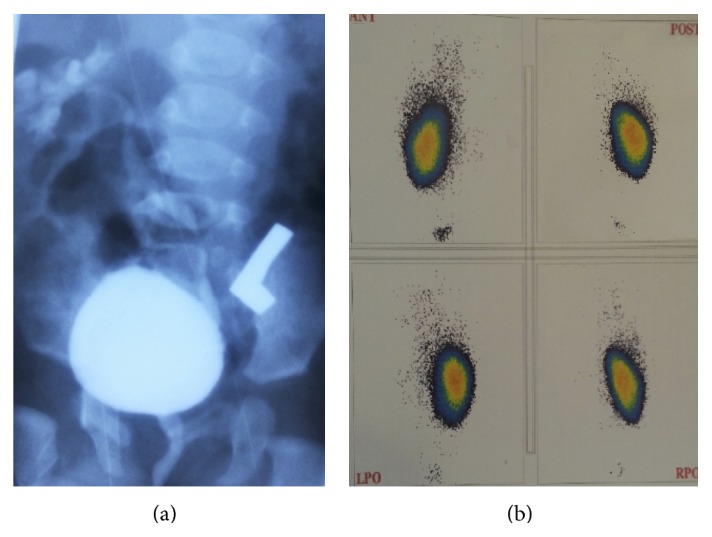
(a) VCUG, anteroposterior view showing VUR; ureter is seen crossing the midline from left to right side at the L5 level. (b) DMSA renal scan demonstrates absent activity in the left renal bed with acceptable cortical function of the right kidney.

A 3-month-old boy born with a single umbilical artery was referred with a penile hypospadias. Ultrasound revealed an empty left renal fossa suggesting renal agenesis and a hypertrophic kidney (2.5 × 7.0 cm) on the right side with normal renal parenchyma. Dimercaptosuccinic acid (DMSA) scan showed acceptable cortical function of the right kidney; no tracer uptake was visualized on the left one. Voiding cystourethrogram (VCUG) demonstrated grade III VUR into the left ureter, with a path crossing the midline and entering the right kidney (Figure 1). On cystoscopy, single ureteral orifice was located on the left side. Dextranomer/hyaluronic acid copolymer (Deflux) was injected at the left ureteral orifice to correct the high-grade VUR. The patient was discharged asymptomatically with the resolution of VUR and was scheduled for a hypospadias repair.

## 3. Case 2

**Figure 2:**
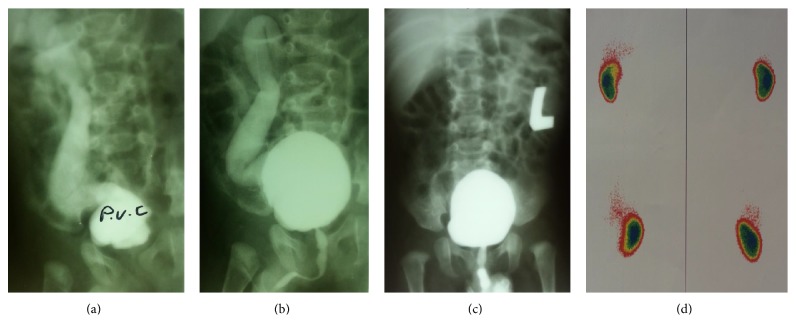
AP and oblique view VCUG showing left to right renal ectopia and high-grade left sided VUR that persisted after first valve ablation ((a), (b)) and resolution of VUR after second valve ablation (c). DMSA renal scan shows proper cortical function of the right kidney and nonvisualization of the left kidney (d).

A 9-day-old male neonate was referred with an antenatal ultrasound suggestive of solitary unilateral hydronephrosis. Postnatal ultrasound confirmed absence of the left kidney; a large right kidney with severe hydroureteronephrosis was reported. DMSA demonstrated absent activity on the left side and normal cortical function of the right kidney. Additionally, initial VCUG revealed VUR into the right kidney and a typical PUV. The patient underwent endoscopic valve ablation at day 15 after birth but the ureteral orifice was not found on the right side. Postoperative VCUG still showed grade IV VUR into the left ureter with a megaureter (diameter: 7.0 mm) that crossed the midline and entered the right kidney. It also showed minimal posterior urethral dilation compatible with PUV remnants. Video urethrocystoscopy confirmed the presence of PUV remnants with a trabeculated bladder and absence of right ureteral orifice. Endoscopic PUV ablation was performed and VUR resolved subsequently in the follow-up VCUG at 6 months of age (Figure 2).

## 4. Discussion

Crossed renal ectopia defines a spectrum of congenital anomalies in which the kidney is located on one side, while the corresponding ureter enters the bladder in the contralateral side. In 1957, McDonald and McClellan [6] revised the categorization of crossed renal ectopia into 4 subtypes: (1) with fusion; (2) without fusion; (3) solitary crossed; and (4) bilateral crossed. Crossed renal ectopia with and without fusion constitutes more than 90% of all cases, whereas solitary crossed renal ectopia (SCRE) and bilateral crossed ectopia are exceedingly rare.

Unilateral renal agenesis accompanied by renal ectopia in the contralateral side results in SCRE. The exact embryologic mechanisms behind this anomaly remain widely unknown. Formation of metanephros begins when the ureteric buds meet the metanephric blastema early in development. Absence or incomplete development of a ureteric bud disrupts the association between collecting and excretory systems and precludes the development of a definitive kidney, being the embryologic basis behind renal agenesis [2]. However, little consensus exists on the exact cause of crossed renal ectopia. Wilmer [7] proposed that pressure from abnormally located umbilical arteries displaces the renal unit and facilitates its ascend to the opposite renal bed, where it faces lesser degrees of mechanical resistance. In another theory, a wandering ureteric bud is the main culprit that joins the contralateral metanephric blastema and continues to ascend in the wrong direction [8]. Ashley and Mostofi [9] also focused on the role of unknown signaling substances produced by contralateral metanephros that attract the developing ureteric bud deviating it from the normal path. Role of teratogens and also misalignment and rotation of medial axis during fetal development are among other theories proposed [2].

Most individuals with SCRE are male (ratio 2 : 1) and have left to right ectopia. SCRE has the highest rate of associated anomalies in crossed ectopia that may be more attributable to renal agenesis rather than crossed ectopia [2]. Absent vas deferens and cryptorchidism in males and vaginal atresia and uterine abnormalities in females are most frequent genitourinary anomalies in this group [2, 5]. Although concomitant VUR is reported in few cases [3, 4], the presence of PUV or hypospadias in SCRE complicated by VUR is exceedingly rare.

In most cases, SCRE remains asymptomatic and is diagnosed incidentally or on autopsy. Proper diagnosis needs a high degree of clinical suspicion and prompt attention to the accompanying abnormalities. Presence of cryptorchidism, vas anomalies, hypospadias, urethral valves, unilateral hydronephrosis, megaureter, or VUR can all signal to the underlying renal anomaly. Vague symptoms may develop later in life as hematuria, pyuria, or abdominal pain [2]. Urinary tract infection or renal calculi may be the only clue and may be attributable to abnormal kidney position or vascular supply that disrupts the normal drainage [2].

In modern medicine, ultrasonography and DMSA have largely replaced classic urography and retrograde pyelograms in diagnosis of SCRE. Although CT-scan and MRI can provide excellent information on urinary tract anatomy, their use is limited by radiation exposure and/or cost. In fact, most of the asymptomatic SCRE patients can be initially diagnosed by ultrasound and DMSA [2]. If needed, VCUG can provide extra information on bladder anatomy, presence of VUR and/or PUV, and path of the refluxing ureter. Cystoscopy also helps in assessing the urethral and bladder anatomy and position of the ureteral orifices or delivering treatment for PUV and VUR if needed. Taken together, ultrasound and DMSA are excellent diagnostic options for SCRE, with CT-scan and MRI reserved for selected cases or before extensive surgeries.

The overall prognosis of SCRE is excellent and most patients have a normal lifespan [2]. Morbidity may be due to associated anomalies needing prompt management. Although Grotas and Phillips [10] recently reported a rare case of renal cell carcinoma in SCRE and suggested an incidence of 1 in 22 million for this condition, even this value may be a large overestimation since they did not use the prevalence of “solitary” crossed renal ectopia in their calculation.

## 5. Summary

In conclusion, this study adds two new cases to the limited literature on SCRE. SCRE is a rare urinary tract disorder that may be asymptomatic or may be accompanied by other skeletal and genitourinary anomalies. Besides previously reported genitourinary comorbidities, hypospadias and PUV should also be regarded as associated anomalies in SCRE patients. It is intuitive to more thoroughly evaluate the urinary tract to find associated conditions when a congenital anomaly is diagnosed.
